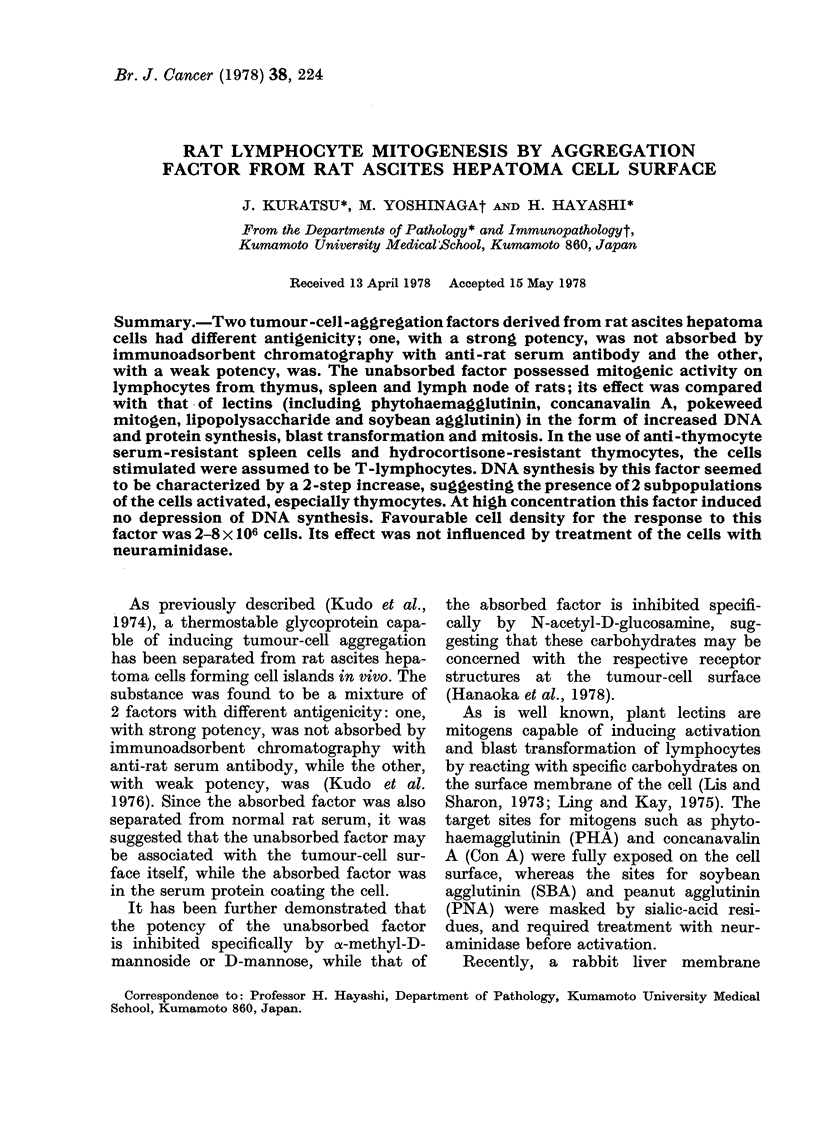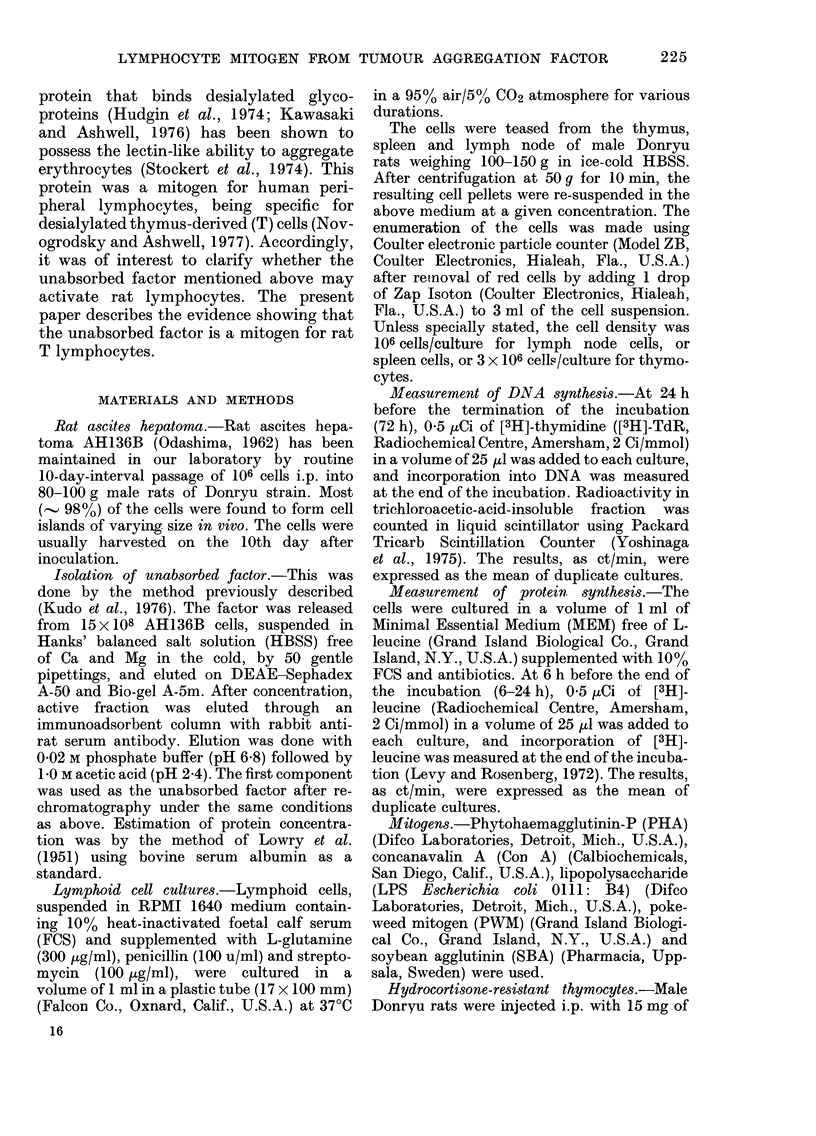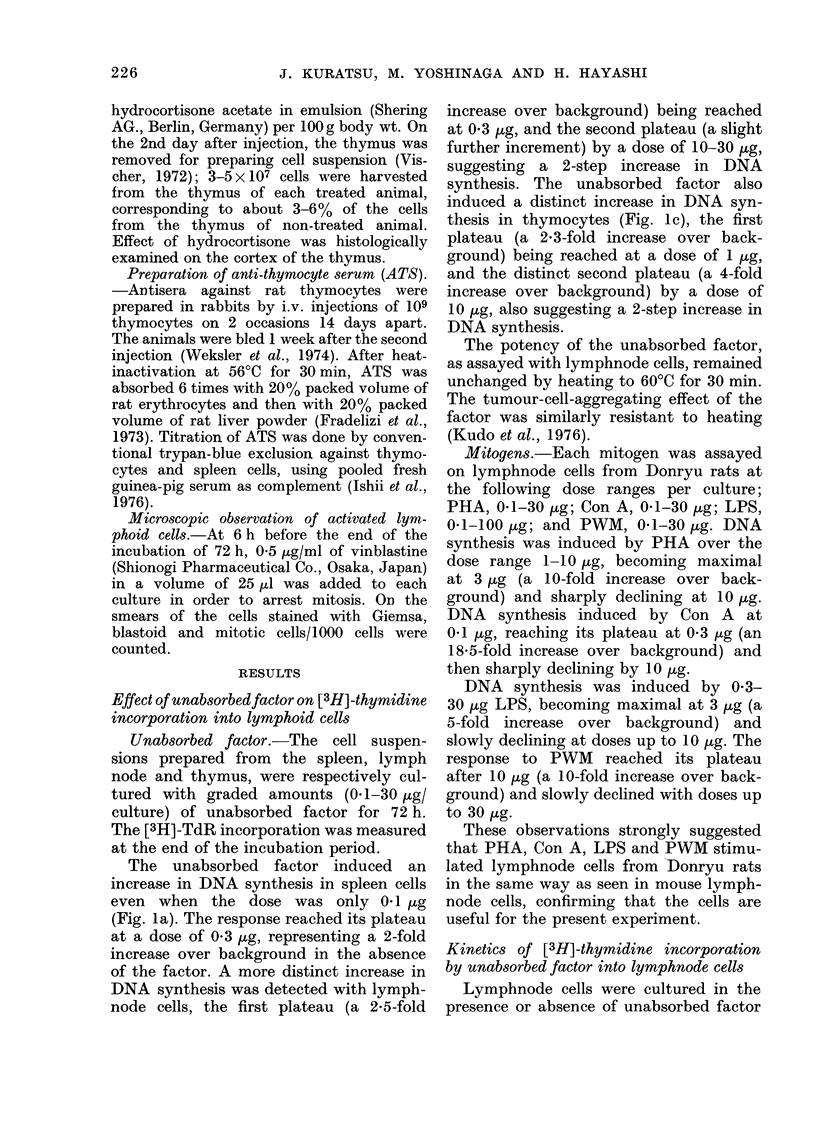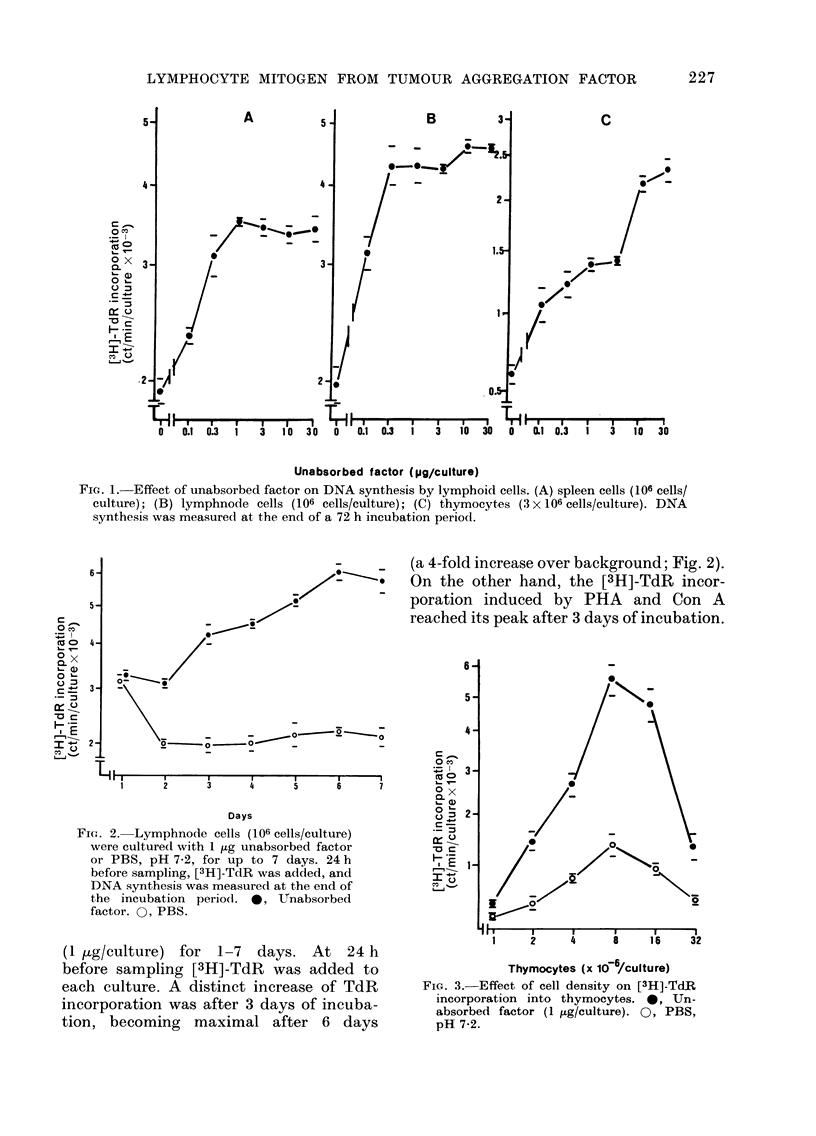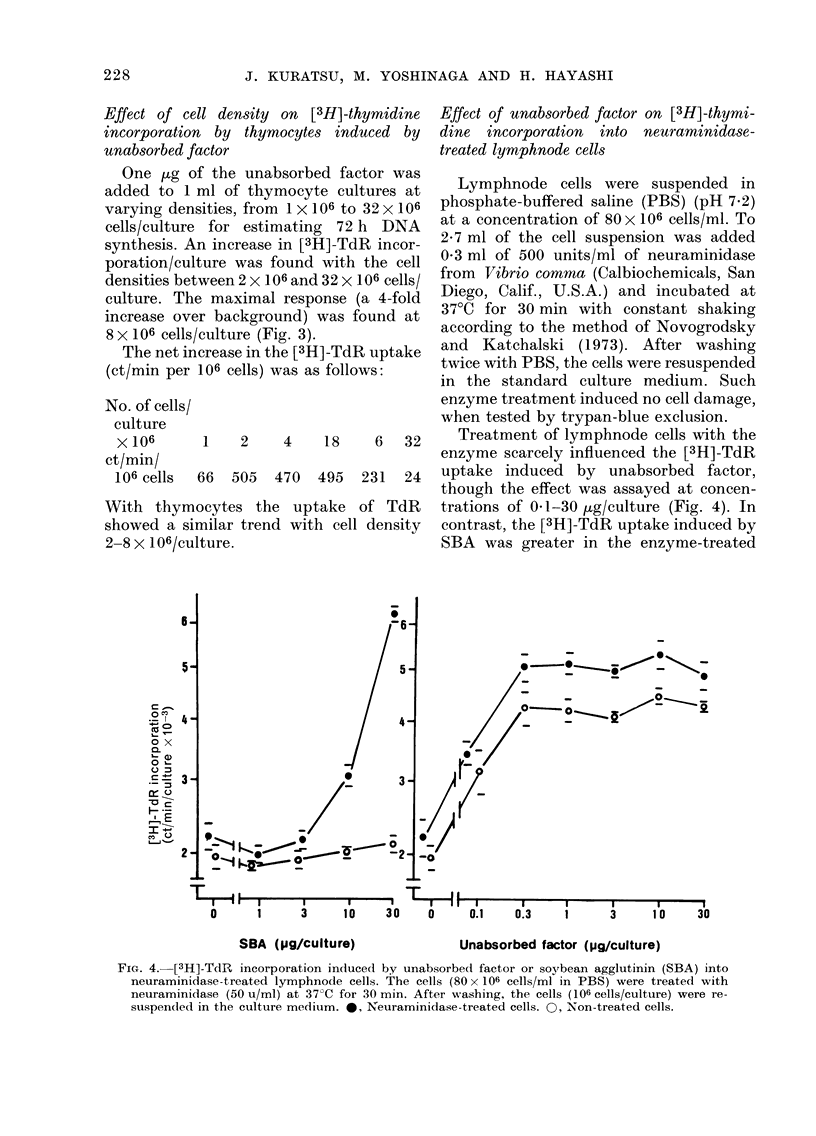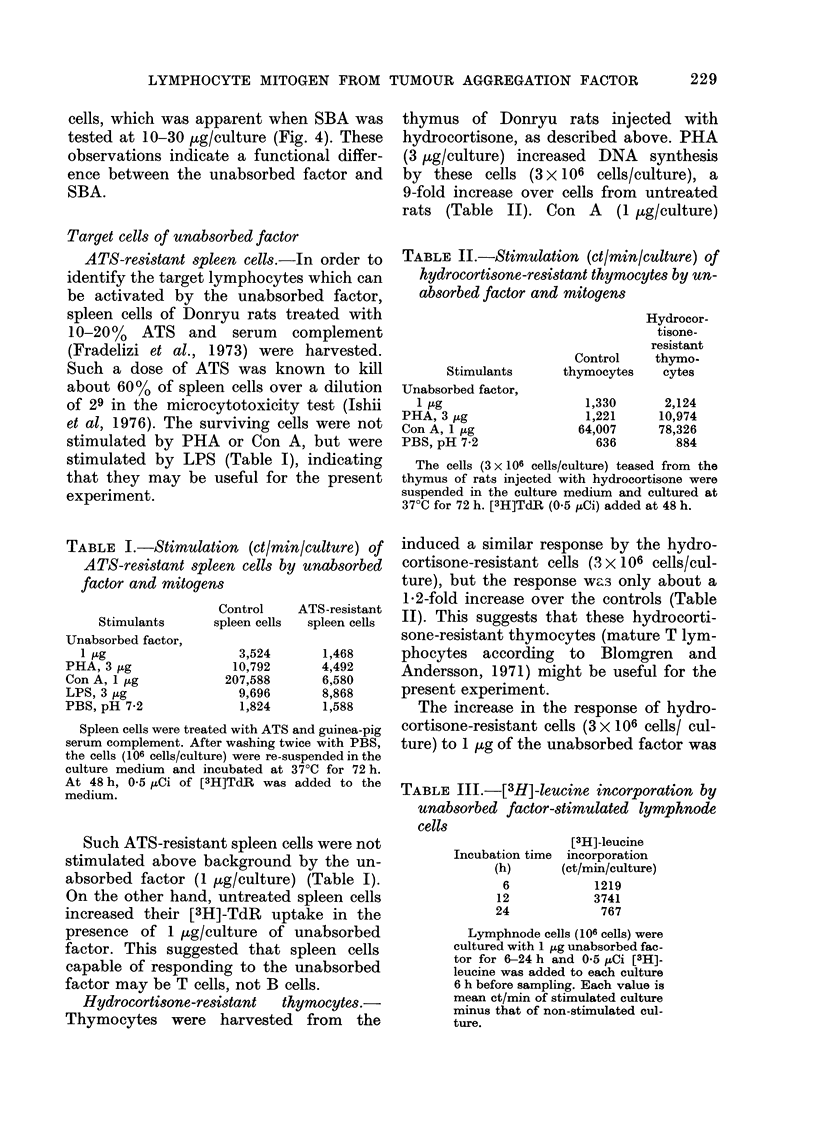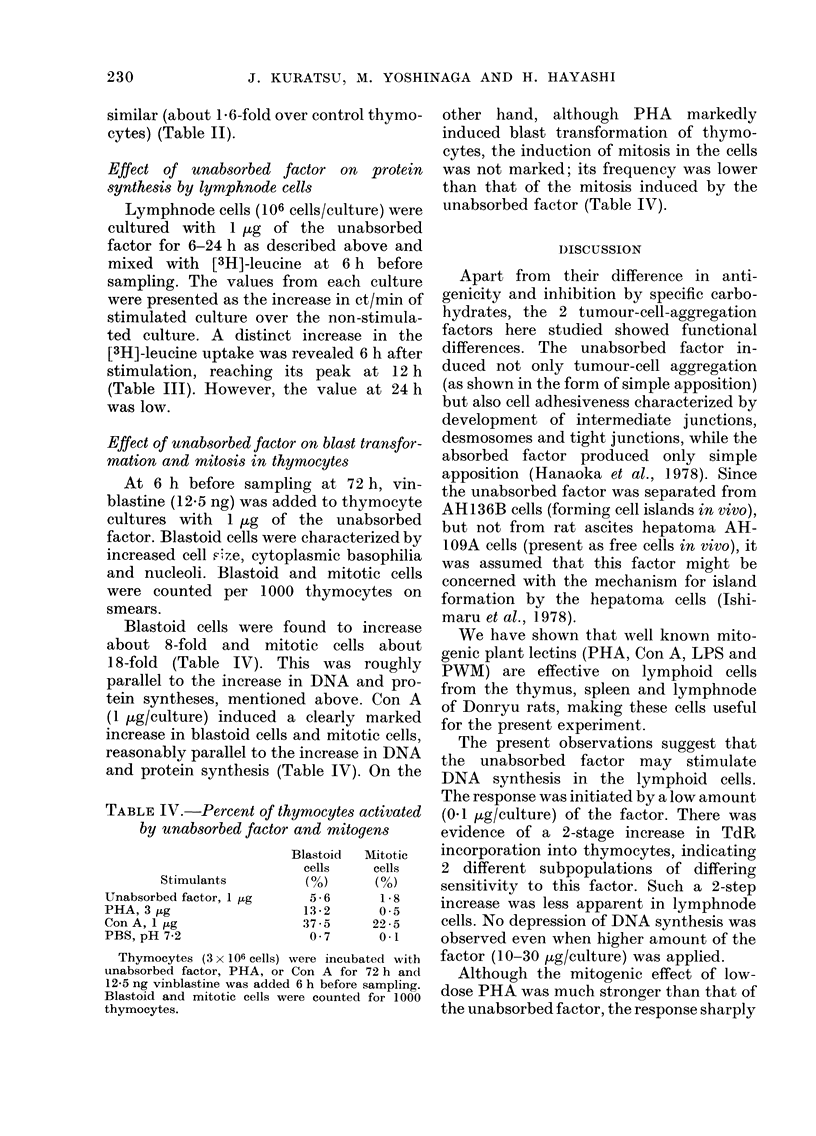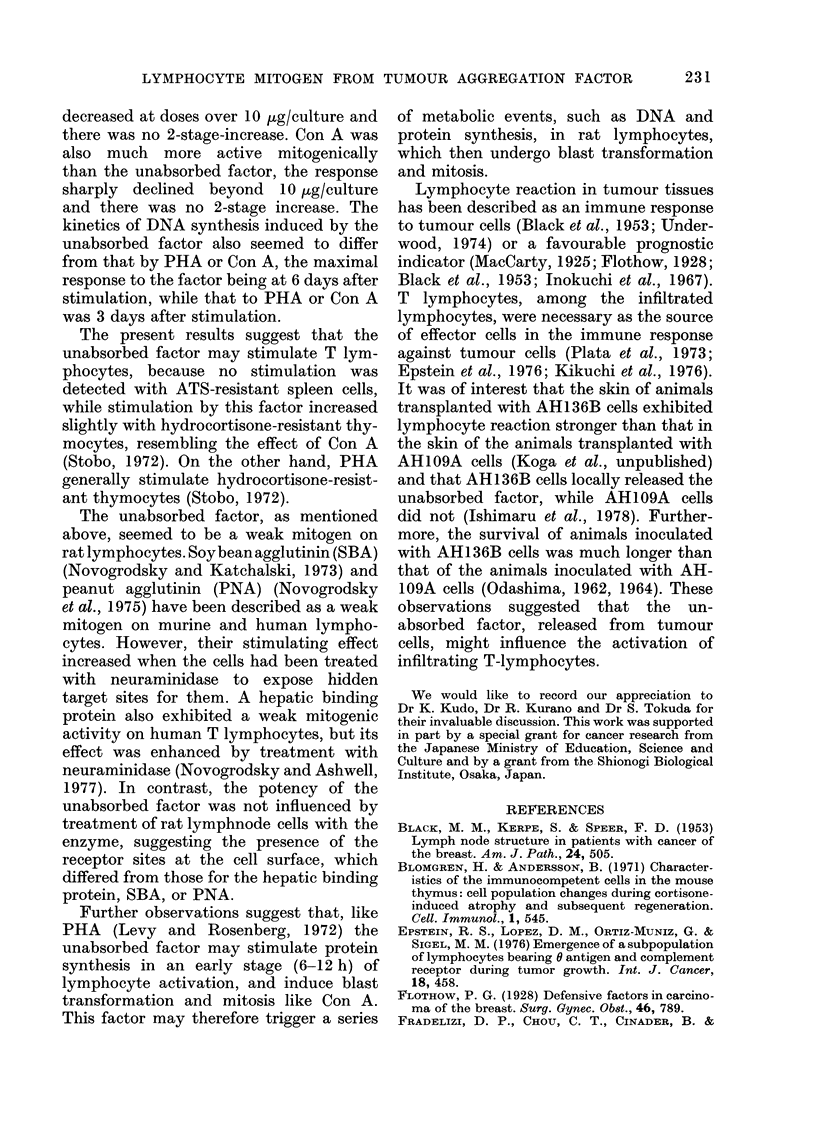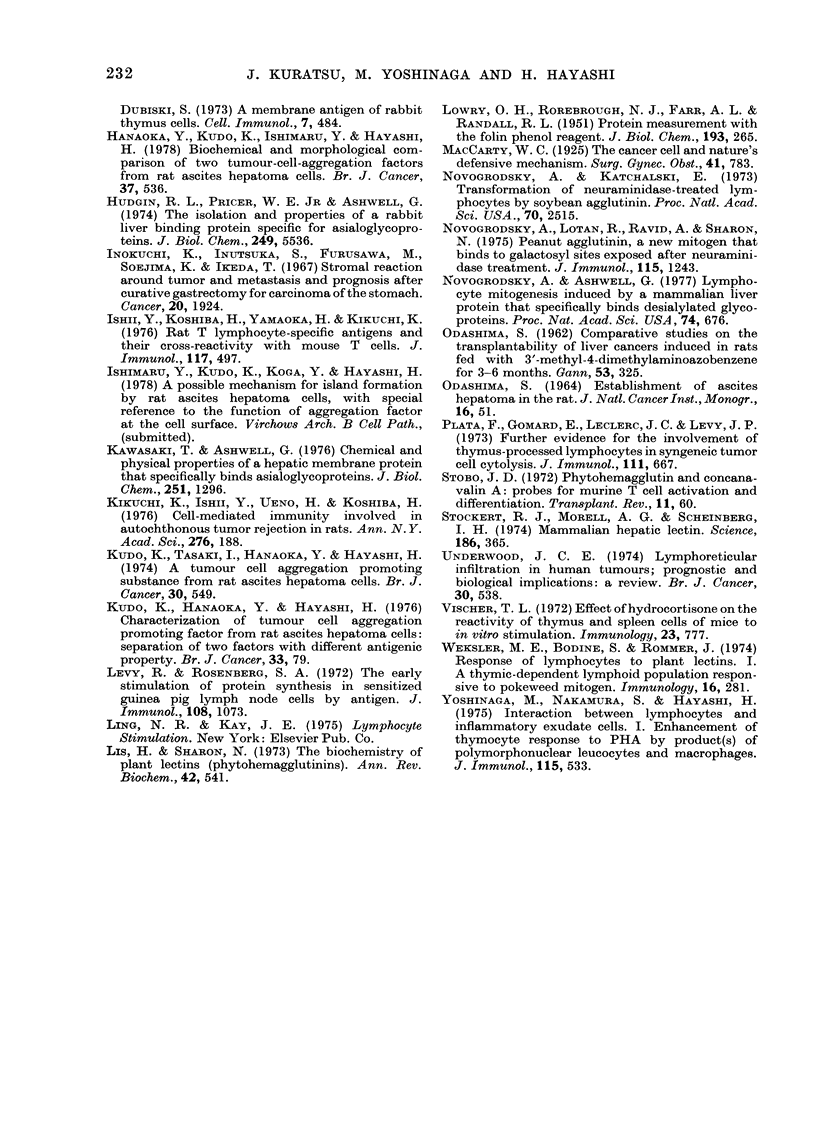# Rat lymphocyte mitogenesis by aggregation factor from rat ascites hepatoma cell surface.

**DOI:** 10.1038/bjc.1978.192

**Published:** 1978-08

**Authors:** J. Kuratsu, M. Yoshinaga, H. Hayashi

## Abstract

Two-tumour-cell-aggregation factors derived from rat ascites hepatoma cells had different antigenicity; one, with a strong potency, was not absorbed by immunoadsorbent chromatography with anti-rat serum antibody and the other, with a weak potency, was. The unabsorbed factor possessed mitogenic activity on lymphocytes from thymus, spleen and lymph node of rats; its effect was compared with that of lectins (including phytohaemagglutinin, concanavalin A, pokeweed mitogen, lipopolysaccharide and soybean agglutinin) in the form of increased DNA and protein synthesis, blast transformation and mitosis. In the use of anti-thymocyte serum-resistant spleen cells and hydrocortisone-resistant thymocytes, the cells stimulated were assumed to be T-lymphocytes. DNA synthesis by this factor seemed to be characterized by a 2-step increase, suggesting the presence of 2 subpopulations of the cells activated, especially thymocytes. At high concentration this factor induced no depression of DNA synthesis. Favourable cell density for the response to this factor was 2-8 X 10(6) cells. Its effect was not influenced by treatment of the cells with neuraminidase.


*Br. J. Cancer* (1978) **38**, 224

### RAT LYMPHOCYTE MITOGENESIS BY AGGREGATION FACTOR FROM RAT ASCITES HEPATOMA CELL SURFACE

J. KURATSU*, M. YOSHINAGA† AND H. HAYASHI*

*From the Departments of Pathology* and Immunopathology†, Kumamoto University Medical School, Kumamoto 860, Japan*

Received 13 April 1978 Accepted 15 May 1978

**Summary.—Two tumour-cell-aggregation factors derived from rat ascites hepatoma cells had different antigenicity; one, with a strong potency, was not absorbed by immunoadsorbent chromatography with anti-rat serum antibody and the other, with a weak potency, was. The unabsorbed factor possessed mitogenic activity on lymphocytes from thymus, spleen and lymph node of rats; its effect was compared with that of lectins (including phytohaemagglutinin, concanavalin A, pokeweed mitogen, lipopolysaccharide and soybean agglutinin) in the form of increased DNA and protein synthesis, blast transformation and mitosis. In the use of anti-thymocyte serum-resistant spleen cells and hydrocortisone-resistant thymocytes, the cells stimulated were assumed to be T-lymphocytes. DNA synthesis by this factor seemed to be characterized by a 2-step increase, suggesting the presence of 2 subpopulations of the cells activated, especially thymocytes. At high concentration this factor induced no depression of DNA synthesis. Favourable cell density for the response to this factor was 2–8 $\times 10^6$ cells. Its effect was not influenced by treatment of the cells with neuraminidase.**

As previously described (Kudo *et al.*, 1974), a thermostable glycoprotein capable of inducing tumour-cell aggregation has been separated from rat ascites hepatoma cells forming cell islands *in vivo*. The substance was found to be a mixture of 2 factors with different antigenicity: one, with strong potency, was not absorbed by immunoadsorbent chromatography with anti-rat serum antibody, while the other, with weak potency, was (Kudo *et al.* 1976). Since the absorbed factor was also separated from normal rat serum, it was suggested that the unabsorbed factor may be associated with the tumour-cell surface itself, while the absorbed factor was in the serum protein coating the cell.

It has been further demonstrated that the potency of the unabsorbed factor is inhibited specifically by $\alpha$-methyl-D-mannoside or D-mannose, while that of the absorbed factor is inhibited specifically by N-acetyl-D-glucosamine, suggesting that these carbohydrates may be concerned with the respective receptor structures at the tumour-cell surface (Hanaoka *et al.*, 1978).

As is well known, plant lectins are mitogens capable of inducing activation and blast transformation of lymphocytes by reacting with specific carbohydrates on the surface membrane of the cell (Lis and Sharon, 1973; Ling and Kay, 1975). The target sites for mitogens such as phytohaemagglutinin (PHA) and concanavalin A (Con A) were fully exposed on the cell surface, whereas the sites for soybean agglutinin (SBA) and peanut agglutinin (PNA) were masked by sialic-acid residues, and required treatment with neuraminidase before activation.

Recently, a rabbit liver membrane

Correspondence to: Professor H. Hayashi, Department of Pathology, Kumamoto University Medical School, Kumamoto 860, Japan.

protein that binds desialylated glycoproteins (Hudgin *et al.*, 1974; Kawasaki and Ashwell, 1976) has been shown to possess the lectin-like ability to aggregate erythrocytes (Stockert *et al.*, 1974). This protein was a mitogen for human peripheral lymphocytes, being specific for desialylated thymus-derived (T) cells (Novogrodsky and Ashwell, 1977). Accordingly, it was of interest to clarify whether the unabsorbed factor mentioned above may activate rat lymphocytes. The present paper describes the evidence showing that the unabsorbed factor is a mitogen for rat T lymphocytes.

### MATERIALS AND METHODS

*Rat ascites hepatoma.*—Rat ascites hepatoma AH136B (Odashima, 1962) has been maintained in our laboratory by routine 10-day-interval passage of $10^6$ cells i.p. into 80–100 g male rats of Donryu strain. Most ($\sim$ 98%) of the cells were found to form cell islands of varying size *in vivo*. The cells were usually harvested on the 10th day after inoculation.

*Isolation of unabsorbed factor.*—This was done by the method previously described (Kudo *et al.*, 1976). The factor was released from $15 \times 10^8$ AH136B cells, suspended in Hanks' balanced salt solution (HBSS) free of Ca and Mg in the cold, by 50 gentle pipettings, and eluted on DEAE–Sephadex A-50 and Bio-gel A-5m. After concentration, active fraction was eluted through an immunoadsorbent column with rabbit anti-rat serum antibody. Elution was done with 0·02 M phosphate buffer (pH 6·8) followed by 1·0 M acetic acid (pH 2·4). The first component was used as the unabsorbed factor after re-chromatography under the same conditions as above. Estimation of protein concentration was by the method of Lowry *et al.* (1951) using bovine serum albumin as a standard.

*Lymphoid cell cultures.*—Lymphoid cells, suspended in RPMI 1640 medium containing 10% heat-inactivated foetal calf serum (FCS) and supplemented with L-glutamine (300 $\mu$g/ml), penicillin (100 u/ml) and streptomycin (100 $\mu$g/ml), were cultured in a volume of 1 ml in a plastic tube ($17 \times 100$ mm) (Falcon Co., Oxnard, Calif., U.S.A.) at 37°C in a 95% air/5% $CO_2$ atmosphere for various durations.

The cells were teased from the thymus, spleen and lymph node of male Donryu rats weighing 100–150 g in ice-cold HBSS. After centrifugation at 50 *g* for 10 min, the resulting cell pellets were re-suspended in the above medium at a given concentration. The enumeration of the cells was made using Coulter electronic particle counter (Model ZB, Coulter Electronics, Hialeah, Fla., U.S.A.) after removal of red cells by adding 1 drop of Zap Isoton (Coulter Electronics, Hialeah, Fla., U.S.A.) to 3 ml of the cell suspension. Unless specially stated, the cell density was $10^6$ cells/culture for lymph node cells, or spleen cells, or $3 \times 10^6$ cells/culture for thymocytes.

*Measurement of DNA synthesis.*—At 24 h before the termination of the incubation (72 h), 0·5 $\mu$Ci of [$^3$H]-thymidine ([$^3$H]-TdR, Radiochemical Centre, Amersham, 2 Ci/mmol) in a volume of 25 $\mu$l was added to each culture, and incorporation into DNA was measured at the end of the incubation. Radioactivity in trichloroacetic-acid-insoluble fraction was counted in liquid scintillator using Packard Tricarb Scintillation Counter (Yoshinaga *et al.*, 1975). The results, as ct/min, were expressed as the mean of duplicate cultures.

*Measurement of protein synthesis.*—The cells were cultured in a volume of 1 ml of Minimal Essential Medium (MEM) free of L-leucine (Grand Island Biological Co., Grand Island, N.Y., U.S.A.) supplemented with 10% FCS and antibiotics. At 6 h before the end of the incubation (6–24 h), 0·5 $\mu$Ci of [$^3$H]-leucine (Radiochemical Centre, Amersham, 2 Ci/mmol) in a volume of 25 $\mu$l was added to each culture, and incorporation of [$^3$H]-leucine was measured at the end of the incubation (Levy and Rosenberg, 1972). The results, as ct/min, were expressed as the mean of duplicate cultures.

*Mitogens.*—Phytohaemagglutinin-P (PHA) (Difco Laboratories, Detroit, Mich., U.S.A.), concanavalin A (Con A) (Calbiochemicals, San Diego, Calif., U.S.A.), lipopolysaccharide (LPS *Escherichia coli* 0111: B4) (Difco Laboratories, Detroit, Mich., U.S.A.), pokeweed mitogen (PWM) (Grand Island Biological Co., Grand Island, N.Y., U.S.A.) and soybean agglutinin (SBA) (Pharmacia, Uppsala, Sweden) were used.

*Hydrocortisone-resistant thymocytes.*—Male Donryu rats were injected i.p. with 15 mg of hydrocortisone acetate in emulsion (Shering AG., Berlin, Germany) per 100 g body wt. On the 2nd day after injection, the thymus was removed for preparing cell suspension (Vischer, 1972); 3–5 $\times 10^7$ cells were harvested from the thymus of each treated animal, corresponding to about 3–6% of the cells from the thymus of non-treated animal. Effect of hydrocortisone was histologically examined on the cortex of the thymus.

*Preparation of anti-thymocyte serum (ATS).*—Antisera against rat thymocytes were prepared in rabbits by i.v. injections of $10^9$ thymocytes on 2 occasions 14 days apart. The animals were bled 1 week after the second injection (Weksler *et al.*, 1974). After heat-inactivation at 56°C for 30 min, ATS was absorbed 6 times with 20% packed volume of rat erythrocytes and then with 20% packed volume of rat liver powder (Fradelizi *et al.*, 1973). Titration of ATS was done by conventional trypan-blue exclusion against thymocytes and spleen cells, using pooled fresh guinea-pig serum as complement (Ishii *et al.*, 1976).

*Microscopic observation of activated lymphoid cells.*—At 6 h before the end of the incubation of 72 h, 0·5 μg/ml of vinblastine (Shionogi Pharmaceutical Co., Osaka, Japan) in a volume of 25 μl was added to each culture in order to arrest mitosis. On the smears of the cells stained with Giemsa, blastoid and mitotic cells/1000 cells were counted.

### RESULTS

### *Effect of unabsorbed factor on [$^3$H]-thymidine incorporation into lymphoid cells*

*Unabsorbed factor.*—The cell suspensions prepared from the spleen, lymph node and thymus, were respectively cultured with graded amounts (0·1–30 μg/culture) of unabsorbed factor for 72 h. The [$^3$H]-TdR incorporation was measured at the end of the incubation period.

The unabsorbed factor induced an increase in DNA synthesis in spleen cells even when the dose was only 0·1 μg (Fig. 1a). The response reached its plateau at a dose of 0·3 μg, representing a 2-fold increase over background in the absence of the factor. A more distinct increase in DNA synthesis was detected with lymphnode cells, the first plateau (a 2·5-fold increase over background) being reached at 0·3 μg, and the second plateau (a slight further increment) by a dose of 10–30 μg, suggesting a 2-step increase in DNA synthesis. The unabsorbed factor also induced a distinct increase in DNA synthesis in thymocytes (Fig. 1c), the first plateau (a 2·3-fold increase over background) being reached at a dose of 1 μg, and the distinct second plateau (a 4-fold increase over background) by a dose of 10 μg, also suggesting a 2-step increase in DNA synthesis.

The potency of the unabsorbed factor, as assayed with lymphnode cells, remained unchanged by heating to 60°C for 30 min. The tumour-cell-aggregating effect of the factor was similarly resistant to heating (Kudo *et al.*, 1976).

*Mitogens.*—Each mitogen was assayed on lymphnode cells from Donryu rats at the following dose ranges per culture; PHA, 0·1–30 μg; Con A, 0·1–30 μg; LPS, 0·1–100 μg; and PWM, 0·1–30 μg. DNA synthesis was induced by PHA over the dose range 1–10 μg, becoming maximal at 3 μg (a 10-fold increase over background) and sharply declining at 10 μg. DNA synthesis induced by Con A at 0·1 μg, reaching its plateau at 0·3 μg (an 18·5-fold increase over background) and then sharply declining by 10 μg.

DNA synthesis was induced by 0·3–30 μg LPS, becoming maximal at 3 μg (a 5-fold increase over background) and slowly declining at doses up to 10 μg. The response to PWM reached its plateau after 10 μg (a 10-fold increase over background) and slowly declined with doses up to 30 μg.

These observations strongly suggested that PHA, Con A, LPS and PWM stimulated lymphnode cells from Donryu rats in the same way as seen in mouse lymphnode cells, confirming that the cells are useful for the present experiment.

### *Kinetics of [$^3$H]-thymidine incorporation by unabsorbed factor into lymphnode cells*

Lymphnode cells were cultured in the presence or absence of unabsorbed factor

FIG. 1.—Effect of unabsorbed factor on DNA synthesis by lymphoid cells. (A) spleen cells ($10^6$ cells/culture); (B) lymphnode cells ($10^6$ cells/culture); (C) thymocytes ($3 \times 10^6$ cells/culture). DNA synthesis was measured at the end of a 72 h incubation period.

FIG. 2.—Lymphnode cells ($10^6$ cells/culture) were cultured with 1 μg unabsorbed factor or PBS, pH 7·2, for up to 7 days. 24 h before sampling, [$^3$H]-TdR was added, and DNA synthesis was measured at the end of the incubation period. ●, Unabsorbed factor. ○, PBS.

(1 μg/culture) for 1–7 days. At 24 h before sampling [$^3$H]-TdR was added to each culture. A distinct increase of TdR incorporation was after 3 days of incubation, becoming maximal after 6 days (a 4-fold increase over background; Fig. 2). On the other hand, the [$^3$H]-TdR incorporation induced by PHA and Con A reached its peak after 3 days of incubation.

FIG. 3.—Effect of cell density on [$^3$H]-TdR incorporation into thymocytes. ●, Unabsorbed factor (1 μg/culture). ○, PBS, pH 7·2.

### *Effect of cell density on [$^3$H]-thymidine incorporation by thymocytes induced by unabsorbed factor*

One $\mu$g of the unabsorbed factor was added to 1 ml of thymocyte cultures at varying densities, from $1\times10^6$ to $32\times10^6$ cells/culture for estimating 72 h DNA synthesis. An increase in [$^3$H]-TdR incorporation/culture was found with the cell densities between $2\times10^6$ and $32\times10^6$ cells/culture. The maximal response (a 4-fold increase over background) was found at $8\times10^6$ cells/culture (Fig. 3).

The net increase in the [$^3$H]-TdR uptake (ct/min per $10^6$ cells) was as follows:

| | | | | | | |
|---|---|---|---|---|---|---|
| No. of cells/ culture $\times10^6$ | 1 | 2 | 4 | 18 | 6 | 32 |
| ct/min/ $10^6$ cells | 66 | 505 | 470 | 495 | 231 | 24 |

With thymocytes the uptake of TdR showed a similar trend with cell density 2–8$\times10^6$/culture.

### *Effect of unabsorbed factor on [$^3$H]-thymidine incorporation into neuraminidase-treated lymphnode cells*

Lymphnode cells were suspended in phosphate-buffered saline (PBS) (pH 7·2) at a concentration of $80\times10^6$ cells/ml. To 2·7 ml of the cell suspension was added 0·3 ml of 500 units/ml of neuraminidase from *Vibrio comma* (Calbiochemicals, San Diego, Calif., U.S.A.) and incubated at 37°C for 30 min with constant shaking according to the method of Novogrodsky and Katchalski (1973). After washing twice with PBS, the cells were resuspended in the standard culture medium. Such enzyme treatment induced no cell damage, when tested by trypan-blue exclusion.

Treatment of lymphnode cells with the enzyme scarcely influenced the [$^3$H]-TdR uptake induced by unabsorbed factor, though the effect was assayed at concentrations of 0·1–30 $\mu$g/culture (Fig. 4). In contrast, the [$^3$H]-TdR uptake induced by SBA was greater in the enzyme-treated

FIG. 4.—[$^3$H]-TdR incorporation induced by unabsorbed factor or soybean agglutinin (SBA) into neuraminidase-treated lymphnode cells. The cells ($80\times10^6$ cells/ml in PBS) were treated with neuraminidase (50 u/ml) at 37°C for 30 min. After washing, the cells ($10^6$ cells/culture) were resuspended in the culture medium. ●, Neuraminidase-treated cells. ○, Non-treated cells.

cells, which was apparent when SBA was tested at 10–30 μg/culture (Fig. 4). These observations indicate a functional difference between the unabsorbed factor and SBA.

*Target cells of unabsorbed factor*

*ATS-resistant spleen cells.*—In order to identify the target lymphocytes which can be activated by the unabsorbed factor, spleen cells of Donryu rats treated with 10–20% ATS and serum complement (Fradelizi *et al.*, 1973) were harvested. Such a dose of ATS was known to kill about 60% of spleen cells over a dilution of $2^9$ in the microcytotoxicity test (Ishii *et al*, 1976). The surviving cells were not stimulated by PHA or Con A, but were stimulated by LPS (Table I), indicating that they may be useful for the present experiment.

TABLE I.—*Stimulation (ct/min/culture) of ATS-resistant spleen cells by unabsorbed factor and mitogens*

| Stimulants | Control spleen cells | ATS-resistant spleen cells |
|---|---|---|
| Unabsorbed factor, 1 μg | 3,524 | 1,468 |
| PHA, 3 μg | 10,792 | 4,492 |
| Con A, 1 μg | 207,588 | 6,580 |
| LPS, 3 μg | 9,696 | 8,868 |
| PBS, pH 7·2 | 1,824 | 1,588 |

Spleen cells were treated with ATS and guinea-pig serum complement. After washing twice with PBS, the cells ($10^6$ cells/culture) were re-suspended in the culture medium and incubated at 37°C for 72 h. At 48 h, 0·5 μCi of [$^3$H]TdR was added to the medium.

Such ATS-resistant spleen cells were not stimulated above background by the unabsorbed factor (1 μg/culture) (Table I). On the other hand, untreated spleen cells increased their [$^3$H]-TdR uptake in the presence of 1 μg/culture of unabsorbed factor. This suggested that spleen cells capable of responding to the unabsorbed factor may be T cells, not B cells.

*Hydrocortisone-resistant thymocytes.*—Thymocytes were harvested from the thymus of Donryu rats injected with hydrocortisone, as described above. PHA (3 μg/culture) increased DNA synthesis by these cells ($3 \times 10^6$ cells/culture), a 9-fold increase over cells from untreated rats (Table II). Con A (1 μg/culture)

TABLE II.—*Stimulation (ct/min/culture) of hydrocortisone-resistant thymocytes by unabsorbed factor and mitogens*

| Stimulants | Control thymocytes | Hydrocortisone-resistant thymocytes |
|---|---|---|
| Unabsorbed factor, 1 μg | 1,330 | 2,124 |
| PHA, 3 μg | 1,221 | 10,974 |
| Con A, 1 μg | 64,007 | 78,326 |
| PBS, pH 7·2 | 636 | 884 |

The cells ($3 \times 10^6$ cells/culture) teased from the thymus of rats injected with hydrocortisone were suspended in the culture medium and cultured at 37°C for 72 h. [$^3$H]TdR (0·5 μCi) added at 48 h.

induced a similar response by the hydrocortisone-resistant cells ($3 \times 10^6$ cells/culture), but the response was only about a 1·2-fold increase over the controls (Table II). This suggests that these hydrocortisone-resistant thymocytes (mature T lymphocytes according to Blomgren and Andersson, 1971) might be useful for the present experiment.

The increase in the response of hydrocortisone-resistant cells ($3 \times 10^6$ cells/ culture) to 1 μg of the unabsorbed factor was

TABLE III.—*[$^3$H]-leucine incorporation by unabsorbed factor-stimulated lymphnode cells*

| Incubation time (h) | [$^3$H]-leucine incorporation (ct/min/culture) |
|---|---|
| 6 | 1219 |
| 12 | 3741 |
| 24 | 767 |

Lymphnode cells ($10^6$ cells) were cultured with 1 μg unabsorbed factor for 6–24 h and 0·5 μCi [$^3$H]-leucine was added to each culture 6 h before sampling. Each value is mean ct/min of stimulated culture minus that of non-stimulated culture.

similar (about 1·6-fold over control thymocytes) (Table II).

*Effect of unabsorbed factor on protein synthesis by lymphnode cells*

Lymphnode cells ($10^6$ cells/culture) were cultured with 1 $\mu$g of the unabsorbed factor for 6–24 h as described above and mixed with [$^3$H]-leucine at 6 h before sampling. The values from each culture were presented as the increase in ct/min of stimulated culture over the non-stimulated culture. A distinct increase in the [$^3$H]-leucine uptake was revealed 6 h after stimulation, reaching its peak at 12 h (Table III). However, the value at 24 h was low.

*Effect of unabsorbed factor on blast transformation and mitosis in thymocytes*

At 6 h before sampling at 72 h, vinblastine (12·5 ng) was added to thymocyte cultures with 1 $\mu$g of the unabsorbed factor. Blastoid cells were characterized by increased cell size, cytoplasmic basophilia and nucleoli. Blastoid and mitotic cells were counted per 1000 thymocytes on smears.

Blastoid cells were found to increase about 8-fold and mitotic cells about 18-fold (Table IV). This was roughly parallel to the increase in DNA and protein syntheses, mentioned above. Con A (1 $\mu$g/culture) induced a clearly marked increase in blastoid cells and mitotic cells, reasonably parallel to the increase in DNA and protein synthesis (Table IV). On the other hand, although PHA markedly induced blast transformation of thymocytes, the induction of mitosis in the cells was not marked; its frequency was lower than that of the mitosis induced by the unabsorbed factor (Table IV).

TABLE IV.—*Percent of thymocytes activated by unabsorbed factor and mitogens*

| Stimulants | Blastoid cells (%) | Mitotic cells (%) |
|---|---|---|
| Unabsorbed factor, 1 $\mu$g | 5·6 | 1·8 |
| PHA, 3 $\mu$g | 13·2 | 0·5 |
| Con A, 1 $\mu$g | 37·5 | 22·5 |
| PBS, pH 7·2 | 0·7 | 0·1 |

Thymocytes ($3 \times 10^6$ cells) were incubated with unabsorbed factor, PHA, or Con A for 72 h and 12·5 ng vinblastine was added 6 h before sampling. Blastoid and mitotic cells were counted for 1000 thymocytes.

### DISCUSSION

Apart from their difference in antigenicity and inhibition by specific carbohydrates, the 2 tumour-cell-aggregation factors here studied showed functional differences. The unabsorbed factor induced not only tumour-cell aggregation (as shown in the form of simple apposition) but also cell adhesiveness characterized by development of intermediate junctions, desmosomes and tight junctions, while the absorbed factor produced only simple apposition (Hanaoka *et al.*, 1978). Since the unabsorbed factor was separated from AH136B cells (forming cell islands *in vivo*), but not from rat ascites hepatoma AH-109A cells (present as free cells *in vivo*), it was assumed that this factor might be concerned with the mechanism for island formation by the hepatoma cells (Ishimaru *et al.*, 1978).

We have shown that well known mitogenic plant lectins (PHA, Con A, LPS and PWM) are effective on lymphoid cells from the thymus, spleen and lymphnode of Donryu rats, making these cells useful for the present experiment.

The present observations suggest that the unabsorbed factor may stimulate DNA synthesis in the lymphoid cells. The response was initiated by a low amount (0·1 $\mu$g/culture) of the factor. There was evidence of a 2-stage increase in TdR incorporation into thymocytes, indicating 2 different subpopulations of differing sensitivity to this factor. Such a 2-step increase was less apparent in lymphnode cells. No depression of DNA synthesis was observed even when higher amount of the factor (10–30 $\mu$g/culture) was applied.

Although the mitogenic effect of low-dose PHA was much stronger than that of the unabsorbed factor, the response sharply decreased at doses over 10 μg/culture and there was no 2-stage-increase. Con A was also much more active mitogenically than the unabsorbed factor, the response sharply declined beyond 10 μg/culture and there was no 2-stage increase. The kinetics of DNA synthesis induced by the unabsorbed factor also seemed to differ from that by PHA or Con A, the maximal response to the factor being at 6 days after stimulation, while that to PHA or Con A was 3 days after stimulation.

The present results suggest that the unabsorbed factor may stimulate T lymphocytes, because no stimulation was detected with ATS-resistant spleen cells, while stimulation by this factor increased slightly with hydrocortisone-resistant thymocytes, resembling the effect of Con A (Stobo, 1972). On the other hand, PHA generally stimulate hydrocortisone-resistant thymocytes (Stobo, 1972).

The unabsorbed factor, as mentioned above, seemed to be a weak mitogen on rat lymphocytes. Soy bean agglutinin (SBA) (Novogrodsky and Katchalski, 1973) and peanut agglutinin (PNA) (Novogrodsky *et al.*, 1975) have been described as a weak mitogen on murine and human lymphocytes. However, their stimulating effect increased when the cells had been treated with neuraminidase to expose hidden target sites for them. A hepatic binding protein also exhibited a weak mitogenic activity on human T lymphocytes, but its effect was enhanced by treatment with neuraminidase (Novogrodsky and Ashwell, 1977). In contrast, the potency of the unabsorbed factor was not influenced by treatment of rat lymphnode cells with the enzyme, suggesting the presence of the receptor sites at the cell surface, which differed from those for the hepatic binding protein, SBA, or PNA.

Further observations suggest that, like PHA (Levy and Rosenberg, 1972) the unabsorbed factor may stimulate protein synthesis in an early stage (6–12 h) of lymphocyte activation, and induce blast transformation and mitosis like Con A. This factor may therefore trigger a series of metabolic events, such as DNA and protein synthesis, in rat lymphocytes, which then undergo blast transformation and mitosis.

Lymphocyte reaction in tumour tissues has been described as an immune response to tumour cells (Black *et al.*, 1953; Underwood, 1974) or a favourable prognostic indicator (MacCarty, 1925; Flothow, 1928; Black *et al.*, 1953; Inokuchi *et al.*, 1967). T lymphocytes, among the infiltrated lymphocytes, were necessary as the source of effector cells in the immune response against tumour cells (Plata *et al.*, 1973; Epstein *et al.*, 1976; Kikuchi *et al.*, 1976). It was of interest that the skin of animals transplanted with AH136B cells exhibited lymphocyte reaction stronger than that in the skin of the animals transplanted with AH109A cells (Koga *et al.*, unpublished) and that AH136B cells locally released the unabsorbed factor, while AH109A cells did not (Ishimaru *et al.*, 1978). Furthermore, the survival of animals inoculated with AH136B cells was much longer than that of the animals inoculated with AH-109A cells (Odashima, 1962, 1964). These observations suggested that the unabsorbed factor, released from tumour cells, might influence the activation of infiltrating T-lymphocytes.

We would like to record our appreciation to Dr K. Kudo, Dr R. Kurano and Dr S. Tokuda for their invaluable discussion. This work was supported in part by a special grant for cancer research from the Japanese Ministry of Education, Science and Culture and by a grant from the Shionogi Biological Institute, Osaka, Japan.

### REFERENCES

Black, M. M., Kerpe, S. & Speer, F. D. (1953) Lymph node structure in patients with cancer of the breast. *Am. J. Path.*, **24**, 505.

Blomgren, H. & Andersson, B. (1971) Characteristics of the immunocompetent cells in the mouse thymus: cell population changes during cortisone-induced atrophy and subsequent regeneration. *Cell. Immunol.*, **1**, 545.

Epstein, R. S., Lopez, D. M., Ortiz-Muniz, G. & Sigel, M. M. (1976) Emergence of a subpopulation of lymphocytes bearing θ antigen and complement receptor during tumor growth. *Int. J. Cancer*, **18**, 458.

Flothow, P. G. (1928) Defensive factors in carcinoma of the breast. *Surg. Gynec. Obst.*, **46**, 789.

Fradelizi, D. P., Chou, C. T., Cinader, B. &

DUBISKI, S. (1973) A membrane antigen of rabbit thymus cells. *Cell. Immunol.*, **7**, 484.

HANAOKA, Y., KUDO, K., ISHIMARU, Y. & HAYASHI, H. (1978) Biochemical and morphological comparison of two tumour-cell-aggregation factors from rat ascites hepatoma cells. *Br. J. Cancer*, **37**, 536.

HUDGIN, R. L., PRICER, W. E. JR & ASHWELL, G. (1974) The isolation and properties of a rabbit liver binding protein specific for asialoglycoproteins. *J. Biol. Chem.*, **249**, 5536.

INOKUCHI, K., INUTSUKA, S., FURUSAWA, M., SOEJIMA, K. & IKEDA, T. (1967) Stromal reaction around tumor and metastasis and prognosis after curative gastrectomy for carcinoma of the stomach. *Cancer*, **20**, 1924.

ISHII, Y., KOSHIBA, H., YAMAOKA, H. & KIKUCHI, K. (1976) Rat T lymphocyte-specific antigens and their cross-reactivity with mouse T cells. *J. Immunol.*, **117**, 497.

ISHIMARU, Y., KUDO, K., KOGA, Y. & HAYASHI, H. (1978) A possible mechanism for island formation by rat ascites hepatoma cells, with special reference to the function of aggregation factor at the cell surface. *Virchows Arch. B Cell Path.*, (submitted).

KAWASAKI, T. & ASHWELL, G. (1976) Chemical and physical properties of a hepatic membrane protein that specifically binds asialoglycoproteins. *J. Biol. Chem.*, **251**, 1296.

KIKUCHI, K., ISHII, Y., UENO, H. & KOSHIBA, H. (1976) Cell-mediated immunity involved in autochthonous tumor rejection in rats. *Ann. N.Y. Acad. Sci.*, **276**, 188.

KUDO, K., TASAKI, I., HANAOKA, Y. & HAYASHI, H. (1974) A tumour cell aggregation promoting substance from rat ascites hepatoma cells. *Br. J. Cancer*, **30**, 549.

KUDO, K., HANAOKA, Y. & HAYASHI, H. (1976) Characterization of tumour cell aggregation promoting factor from rat ascites hepatoma cells: separation of two factors with different antigenic property. *Br. J. Cancer*, **33**, 79.

LEVY, R. & ROSENBERG, S. A. (1972) The early stimulation of protein synthesis in sensitized guinea pig lymph node cells by antigen. *J. Immunol.*, **108**, 1073.

LING, N. R. & KAY, J. E. (1975) *Lymphocyte Stimulation.* New York: Elsevier Pub. Co.

LIS, H. & SHARON, N. (1973) The biochemistry of plant lectins (phytohemagglutinins). *Ann. Rev. Biochem.*, **42**, 541.

LOWRY, O. H., ROREBROUGH, N. J., FARR, A. L. & RANDALL, R. L. (1951) Protein measurement with the folin phenol reagent. *J. Biol. Chem.*, **193**, 265.

MACCARTY, W. C. (1925) The cancer cell and nature's defensive mechanism. *Surg. Gynec. Obst.*, **41**, 783.

NOVOGRODSKY, A. & KATCHALSKI, E. (1973) Transformation of neuraminidase-treated lymphocytes by soybean agglutinin. *Proc. Natl. Acad. Sci. USA.*, **70**, 2515.

NOVOGRODSKY, A., LOTAN, R., RAVID, A. & SHARON, N. (1975) Peanut agglutinin, a new mitogen that binds to galactosyl sites exposed after neuraminidase treatment. *J. Immunol.*, **115**, 1243.

NOVOGRODSKY, A. & ASHWELL, G. (1977) Lymphocyte mitogenesis induced by a mammalian liver protein that specifically binds desialylated glycoproteins. *Proc. Nat. Acad. Sci. USA*, **74**, 676.

ODASHIMA, S. (1962) Comparative studies on the transplantability of liver cancers induced in rats fed with 3′-methyl-4-dimethylaminoazobenzene for 3–6 months. *Gann*, **53**, 325.

ODASHIMA, S. (1964) Establishment of ascites hepatoma in the rat. *J. Natl. Cancer Inst., Monogr.*, **16**, 51.

PLATA, F., GOMARD, E., LECLERC, J. C. & LEVY, J. P. (1973) Further evidence for the involvement of thymus-processed lymphocytes in syngeneic tumor cell cytolysis. *J. Immunol.*, **111**, 667.

STOBO, J. D. (1972) Phytohemagglutin and concanavalin A: probes for murine T cell activation and differentiation. *Transplant. Rev.*, **11**, 60.

STOCKERT, R. J., MORELL, A. G. & SCHEINBERG, I. H. (1974) Mammalian hepatic lectin. *Science*, **186**, 365.

UNDERWOOD, J. C. E. (1974) Lymphoreticular infiltration in human tumours; prognostic and biological implications: a review. *Br. J. Cancer*, **30**, 538.

VISCHER, T. L. (1972) Effect of hydrocortisone on the reactivity of thymus and spleen cells of mice to *in vitro* stimulation. *Immunology*, **23**, 777.

WEKSLER, M. E., BODINE, S. & ROMMER, J. (1974) Response of lymphocytes to plant lectins. I. A thymic-dependent lymphoid population responsive to pokeweed mitogen. *Immunology*, **16**, 281.

YOSHINAGA, M., NAKAMURA, S. & HAYASHI, H. (1975) Interaction between lymphocytes and inflammatory exudate cells. I. Enhancement of thymocyte response to PHA by product(s) of polymorphonuclear leucocytes and macrophages. *J. Immunol.*, **115**, 533.